# Squamoid eccrine ductal carcinoma: series of five cases of a rare tumor^☆^

**DOI:** 10.1016/j.abd.2023.10.006

**Published:** 2024-08-05

**Authors:** Cecília Mirelle Almeida Honorato, Giovanna Gelli Carrascoza, Nubia Marrer Abed, Fernanda Gonçalves Moya

**Affiliations:** Department of Dermatology, Faculty of Medicine, Hospital das Clínicas, Universidade de São Paulo, São Paulo, SP, Brazil

Dear Editor,

Squamoid eccrine ductal carcinoma (SEDC) is a rare malignant cutaneous neoplasm that is biphasic on histopathology, with both squamous (tumor surface) and ductal eccrine (tumor depth) differentiation, often being confused with squamous cell carcinoma (SCC), especially in superficial biopsies. SDEC has significant clinical relevance due to its potential for metastasis and local aggressiveness.^1^, ^2^ Therefore, the objective of this case series is to demonstrate the clinical and histopathological presentation of five cases of SEDC aiming to improve knowledge and management of this rare neoplasm.

Table 1 summarizes the main information of the five SEDC cases. The disease affected exclusively elderly male adults, averaged 68 years old. All cases occurred in the head and neck region, with a predilection for the face (three of five cases). There was a history of immunosuppression due to organ transplantation in three patients and previous local radiotherapy in one case. On histopathology (Fig. 1), the tumors showed an infiltrative growth pattern in the dermis, occasionally invading the subcutaneous and muscular tissue. In the most superficial regions of the tumors, squamous differentiation was observed, similar to well-differentiated SCC, while in the deeper regions, there were different degrees of ductal differentiation and nests and cords of epithelial cells with moderate to severe atypia, surrounded by desmoplastic stroma. The presence of perineural invasion was observed in three cases, and angiolymphatic invasion in one case. Ductal differentiation was confirmed by immunohistochemistry positivity for epithelial membrane antigen (EMA) and carcinoembryonic antigen (CEA) in all tumors (Fig. 2). Information on treatment and evolution is available for four of the five patients, as one of them was lost to follow-up after incisional biopsy of the tumor. All four cases underwent surgical treatment with free surgical margins after the procedure. The surgical margins were evaluated intraoperatively by frozen sections in two of the four cases, while in the other two cases, margin analysis occurred after surgery. The mean follow-up time after surgery was 28.5 months. Evidence of local recurrence was observed in two patients: one had undergone conventional surgery with margin analysis, while the other had intraoperative margin analysis performed using frozen sections. Moreover, in addition to developing local recurrence, one of the patients also showed lymph node and lung metastases, resulting in death.

**Table 1:** Summary of cases of squamoid eccrine ductal carcinoma.

	**Gender**	**Age (years)**	**Site of the tumor**	**Immunosuppression**	**Presence of perineural and angiolymphatic invasion**	**Treatment**	**Time of follow-up (months)**	**Outcome**
**1**	M	72	Eyebrow	Kidney transplant recipient using tacrolimus, azathioprine and prednisone	Perineural invasion	Not applicable	Does not apply	Lost to follow-up
**2**	M	60	Temporal region	Kidney transplant recipient using tacrolimus and everolimus	Perineural and angiolymphatic invasion	Surgical excision with intraoperative margin control by frozen sections	35	No signs of local recurrence or metastasis
**3**	M	73	Forehead	No	No	Conventional surgical excision	21	No signs of local recurrence or metastasis
**4**	M	73	Cervical region	No	No	Conventional surgical excision	36	Local recurrence
**5**	M	62	Scalp	Kidney transplant recipient using prednisone	Perineural invasion	Surgical excision with intraoperative margin control by frozen sections	22	Local recurrence and lung and lymph node metastasis, resulting in death

M: Male.

**Figure 1:**
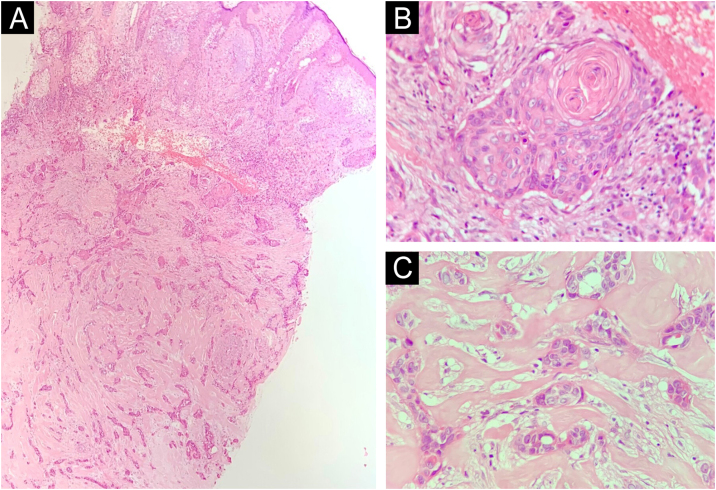
Histopathology. Histological section showing tumor with infiltrative growth invading the deep dermis (A: Hematoxylin & eosin, ×4), with squamous differentiation in the tumor surface (B: Hematoxylin & eosin, ×20) and eccrine ductal differentiation in the tumor depth (C: Hematoxylin & eosin, ×10).

**Figure 2:**
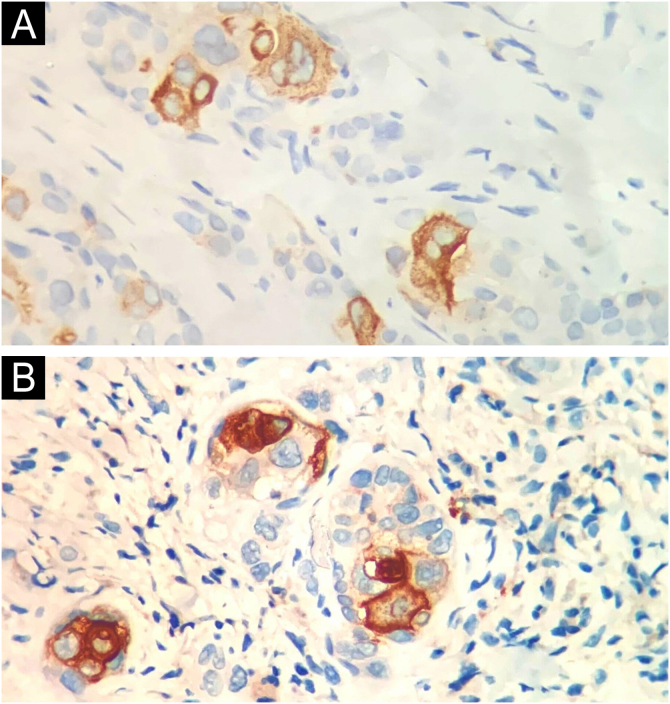
Immunohistochemistry positivity for EMA (A) and CEA (B), confirming the presence of ductal differentiation.

SEDC is traditionally classified as a subtype of eccrine carcinoma. However, its etiopathogenesis remains uncertain, as it is not clear whether it arises from the eccrine ducts with subsequent squamoid differentiation, whether it is an SCC subtype, or whether it is a truly hybrid tumor. SEDC mainly affects the elderly, generally in the seventh or eighth decade of life, and it is suggested that immunosuppression may be a risk factor. Most cases are observed on sun-damaged skin, especially on the head and neck, with the face being the most common location. Clinically, it appears as small, often ulcerated, nodules and plaques, as shown in Fig. 3.^1^, ^2^, ^3^

**Figure 3:**
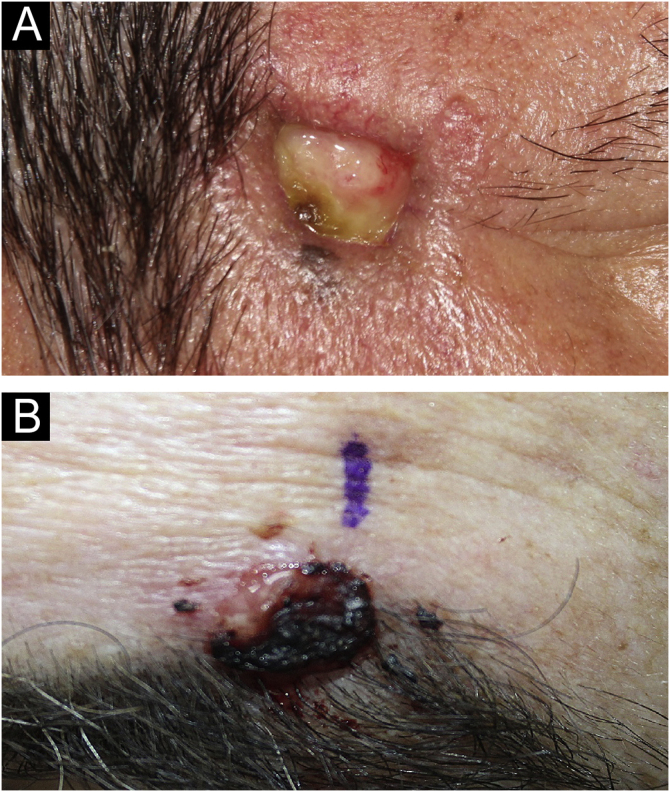
Clinical aspect. (A) Normochromic plaque, measuring approximately 2 cm, with irregular vessels at the border and an ulcerated center. (B) Erythematous nodule, measuring approximately 2 cm, presenting a friable surface covered by an hematic crust.

The diagnosis is made through anatomopathological examination, in which the SEDC presents as a biphasic tumor. In the superficial areas of the tumor, squamous differentiation occurs with connection to the epidermis, while in deeper areas there is clear ductal eccrine differentiation. Squamous differentiation is typically absent in deeper regions. Furthermore, infiltrative growth is observed, with the presence of cords of cytologically atypical epithelial cells, as well as a surrounding desmoplastic stromal response.^1^, ^2^, ^3^, ^4^, ^5^ SEDC frequently extends to the subcutaneous tissue and may be associated with perineural and angiolymphatic invasion, factors that may explain its high local recurrence rate (25%), even after complete excision, and its potential for metastasis (13%), according to literature data.^1^ SEDC demands involves wide local excision and regular clinical follow-up, with Mohs micrographic surgery as a beneficial option.^1^

Therefore, given the rarity and lack of knowledge associated with this neoplasm, together with its potential for unfavorable outcomes, it is essential to conduct additional studies to expand the understanding and management of SEDC. The present findings suggest the need for intensified surveillance of kidney transplant recipients, as three of the five cases occurred in this group of patients. Raising awareness about this neoplasm is essential, as it is probably underdiagnosed, aiming to ensure its early diagnosis and adequate treatment and, consequently, improve clinical outcome.

## Financial support

None declared.

## Authors contributions

Cecília Mirelle Almeida Honorato: Design and planning of the study; drafting and editing of the manuscript; collection, analysis, and interpretation of data; critical review of the literature.

Giovanna Gelli Carrascoza: Drafting and editing of the manuscript; collection, analysis, and interpretation of data; critical review of the literature.

Nubia Marrer Abed: Approval of the final version of the manuscript; intellectual participation in the propaedeutic and/or therapeutic conduct of the studied cases; critical review of the manuscript.

Fernanda Gonçalves Moya: Approval of the final version of the manuscript; design and planning of the study; effective participation in research orientation; critical review of the manuscript.

## Conflicts of interest

None declared.
